# Extracellular matrix substrates differentially influence enteric glial cell homeostasis and immune reactivity

**DOI:** 10.3389/fimmu.2024.1401751

**Published:** 2024-07-25

**Authors:** Linda Schneider, Reiner Schneider, Ebrahim Hamza, Sven Wehner

**Affiliations:** Department of Surgery, Medical Faculty, University Hospital Bonn, Bonn, Germany

**Keywords:** enteric glia, intestinal immune response, extracellular matrix, neuroinflammation, Matrigel, laminin

## Abstract

**Introduction:**

Enteric glial cells are important players in the control of motility, intestinal barrier integrity and inflammation. During inflammation, they switch into a reactive phenotype enabling them to release inflammatory mediators, thereby shaping the inflammatory environment. While a plethora of well-established *in vivo* models exist, cell culture models necessary to decipher the mechanistic pathways of enteric glial reactivity are less well standardized. In particular, the composition of extracellular matrices (ECM) can massively affect the experimental outcome. Considering the growing number of studies involving primary enteric glial cells, a better understanding of their homeostatic and inflammatory *in vitro* culture conditions is needed.

**Methods:**

We examined the impact of different ECMs on enteric glial culture purity, network morphology and immune responsiveness. Therefore, we used immunofluorescence and brightfield microscopy, as well as 3’ bulk mRNA sequencing. Additionally, we compared cultured cells with *in vivo* enteric glial transcriptomes isolated from Sox10^iCreERT2^Rpl22^HA/+^ mice.

**Results:**

We identified Matrigel and laminin as superior over other coatings, including poly-L-ornithine, different lysines, collagens, and fibronectin, gaining the highest enteric glial purity and most extended glial networks expressing connexin-43 hemichannels allowing intercellular communication. Transcriptional analysis revealed strong similarities between enteric glia on Matrigel and laminin with enrichment of gene sets supporting neuronal differentiation, while cells on poly-L-ornithine showed enrichment related to cell proliferation. Comparing cultured and *in vivo* enteric glial transcriptomes revealed a 50% overlap independent of the used coating substrates. Inflammatory activation of enteric glia by IL-1β treatment showed distinct coating-dependent gene expression signatures, with an enrichment of genes related to myeloid and epithelial cell differentiation on Matrigel and laminin coatings, while poly-L-ornithine induced more gene sets related to lymphocyte differentiation.

**Discussion:**

Together, changes in morphology, differentiation and immune activation of primary enteric glial cells proved a strong effect of the ECM. We identified Matrigel and laminin as pre-eminent substrates for murine enteric glial cultures. These new insights will help to standardize and improve enteric glial culture quality and reproducibility between *in vitro* studies in the future, allowing a better comparison of their functional role in enteric neuroinflammation.

## Introduction

Enteric glia are a well-integrated cell type of the enteric nervous system (ENS), and they are closely intertangled with enteric neurons and resident macrophages within the bowel wall. Recent studies have shown that enteric glia are major contributors to intestinal inflammation (1). Driven by mediators, actively or passively released during inflammation, trauma or even stress, enteric glia switch into a so-called reactive phenotype, allowing them to actively participate in the inflammation and control the intestinal immune environment. The molecules able to induce glial reactivity include extracellular ATP (2), IFNγ (3), endothelin-B (4), LPS (5, 6) and interleukin-1 (IL-1). The latter is one of the most-studied inducers of enteric glial reactivity in acute (7–9) and chronic inflammation (10–12). At the cellular level, resident macrophages (7, 11, 13), as well as infiltrating monocyte-derived macrophages (7, 9), have been shown to communicate with enteric glia during gut inflammation.

Besides a plethora of *in vivo* models, including glial-specific reporter and knockout mice, *in vitro* experiments were part of most enteric glia-related studies and have contributed largely to our current understanding of reactive glial biology, their intercellular communication and enteric glia-to-neuron signaling under physiological conditions (14). Meanwhile, most studies use primary enteric glial cultures instead of the few available enteric glial cell lines, such as CRL-2690 (15) and ClK (16). However, primary cultures have certain challenges and are far away from being used in a standardized manner. One of these challenges is the glial cell isolation from intestinal tissue. Most enteric glia are well orchestrated in ganglia and as extraganglionic fibers. Due to their well-integrated phenotype, the main problem is that excessive enzymatic tissue digestion, used to achieve single-cell suspension for *in vitro* studies, also severely disrupts their stellate projections and the extracellular matrix (ECM) of enteric glia and finally leads to cell death. Therefore, researchers developed methods to culture incompletely digested ganglia or so-called neurospheres, leaving the ganglionic glia mostly intact. Both allow the outgrowth and proliferation of enteric glia from the ganglia or neurospheres (17). The second underestimated factor in enteric glial cultures is the contamination by non-glial cell types, which can also grow out of the cultured structures. Depending on the culture conditions, these cells might grow faster and better than glia or at least “contaminate” the glia cultures to a certain degree.

A major factor influencing primary cell quality but also cellular behavior is the choice of the appropriate ECM compounds, as shown for enteric neural-crest-derived cells (18) (giving birth to enteric neurons and glia) and CNS cells (19). In the ENS, the ECM not only favors adhesion and growth of glia and neurons but also affects their cellular functions. For example, enteric neuronal crest cell migration along the developmental gut is strongly controlled by the ECM (18). Furthermore, fibronectin and laminin (20, 21), with the latter particularly expressed by fibroblast and smooth muscle cells in the bowel wall, prevented neuronal differentiation and favored GFAP^+^ enteric glial cell differentiation in ENS cultures. Laminin (21) as well as collagen type IV and fibronectin, among others, were shown to be part of the basement membrane of enteric ganglia, but are not expressed insight the cells (20). A comparative study using various ECM coatings, including laminin, collagens, poly-lysine and heparan sulfate, found different effects on neuronal or glial differentiation (22). Additionally, macrophages, which lay in close spatial proximity to enteric glia in the intestine and can interact and communicate with them (7, 9, 13), have been shown to also be significantly affected by the ECM composition (23, 24). We hypothesized that the ECM also might influence the reactive behavior of enteric glia, one of their most important functions in intestinal diseases.

In this study, we tested the impact of various ECM compounds on the growth, network capacity, and neuronal differentiation, as well as their impact on the immune reactivity of IL-1β-stimulated reactive enteric glia. We discovered striking differences in cell numbers, intercellular networking and differentiation capacities, and differences in the transcriptional response of IL-1β-stimulated reactive enteric glia.

## Materials and methods

### Animals

8- to 16 weeks-old mice of the following lines were used in the study: C57BL/6J (JAX:000664), GFAP^Cre^Ai14^fl/fl^ (derived by crossing B6.Cg-Tg(Gfap-cre)73.12Mvs/J (JAX:012886) and B6;129S6-Gt(ROSA)26Sor^tm14(CAG-tdTomato)Hze^/J (JAX:007908)) or Sox10^iCreERT2^Rpl22^HA/+^ (B6-Tg(Sox10-icre/ERT2)388Wdr/J (Sox10^iCreERT2^; kindly provided by Dr. Vassilis Pachnis (The Francis Crick Institute, London, UK) (25)) and B6N.129-Rpl22^tm1.1Psam^/J (JAX:011029)). C57BL6 and GFAP^Cre^Ai14^fl/fl^ mice were used for primary enteric glial cell isolation, and Sox10^iCreERT2^Rpl22^HA/+^ was used for glial-specific mRNA isolation from intestinal tissue via the *RiboTag* approach. Sox10^iCreERT2^Rpl22^HA/+^ were injected intraperitoneally with 100 µl Tamoxifen (MP Biomedicals, 1 mg in 100 μL sterile corn oil) on three consecutive days and rested for at least one week before organ harvesting. All experiments were carried out under German federal law of North Rhine-Westphalia (AZ 81-02.04.2021.A424).

### Neurosphere-derived enteric glial cell cultures

Primary enteric glial cells were isolated from small intestine *muscularis externa* as described previously (2). Briefly, small intestines of 8-16 weeks old mice were harvested, flushed with cold Krebs-Henseleit buffer (126 mM NaCl; 2.5 mM KCl: 25 mM NaHCO3; 1.2 mM NaH2PO4; 1.2 mM MgCl2; 2.5 mM CaCl2, 100 IU/mL Penicillin, 100 IU/mL Streptomycin and 2.5 μg/mL Amphotericin), cut into segments and kept in oxygenated Krebs-Henseleit buffer on ice. *Muscularis externa* was peeled and collected for digestion. Tissues were digested for 15 min in DMEM containing protease Type 1 (0.25 mg/mL, Sigma-Aldrich) and collagenase A (1 mg/mL, Sigma-Aldrich) at 37°C and 130 rpm. DMEM containing 10% FBS (Sigma-Adrich) was used to stop the enzymatic reaction, and cells were pelleted at 300xg for 5 min. Cell pellets were resuspended in proliferation medium (neurobasal medium with 100 IU/Penicillin, 100 μg/mL Streptomycin, 2.5 μg/mL Amphotericin (all ThermoFisher Scientific), FGF and EGF (both 20 ng/mL, Immunotools)) and cultured on uncoated plates for seven days at 37°C and 5% CO2, applying fresh growth factors every other day. Neurospheres were collected after one week of proliferation and dissociated with trypsin (0.25%, ThermoFisher Scientific) for 5 min at 37°C prior to seeding on coated cover glasses in 24-well plates for differentiation (coating procedures described separately). Neurospheres were kept in differentiation medium (neurobasal medium with 100 IU/Penicillin, 100 μg/mL Streptomycin, 2.5 μg/mL Amphotericin, B27, N2 (all Thermo Scientific) and EGF (2 ng/mL, Immunotools)) for three days for morphological analysis, or five days for further analyses. For IL-1β stimulation, cells were treated with or without IL-1β (10 ng/mL, Immunotools) for 24 hours on day five of differentiation. Supernatants were collected, and cells were lysed with RLT buffer (Qiagen) to isolate mRNA. RNA samples were extracted using the RNeasy Mini Kit (Qiagen) according to the manufacturer’s instructions.

### Coating substrates

Working solutions for coating substrates were prepared as recommended by the manufacturer, as stated in **Table 1**. Cover glasses in 24-well cell culture plates were incubated with 475 µl/well of coating substrates at 37°C overnight and washed according to the respective manufacturer’s instructions before cell seeding (**Table 1**).

**Table 1:** Coating substrates and preparation details.

Coating substrate	Company	Working concentration			Medium	Washing steps	Drying
		lower	standard	higher			
Matrigel	Corning	20 µg/ml (= 25 µg/cm²)	100 µg/ml (= 25 µg/cm²)	250 µg/ml (= 25 µg/cm²)	serum-free medium	no wash	no
Laminin	R&D Systems	4 µg/ml (= 5 µg/cm²)	20 µg/ml (= 5 µg/cm²)	50 µg/ml (= 5 µg/cm²)	serum-free medium	no wash	no
Poly-L-ornithine	Sigma	1.6 µg/ml (= 2 µg/cm²)	8 µg/ml (= 2 µg/cm²)	20 µg/ml (= 2 µg/cm²)	ddH2O	3x ddH2O	no
Poly-L-lysine	Thermo Fisher Scientific	–	8 µg/ml (= 2 µg/cm²)	–	ddH2O	3x ddH2O	no
Poly-D-lysine	ScienCell	–	32 µg/ml (= 8 µg/cm²)	–	PBS	3x ddH2O	yes
Collagen I	Thermo Fisher Scientific	–	20 µg/ml (= 5 µg/cm²)	–	20 mM acetic acid	3x PBS	no
Collagen IV	Corning	–	20 µg/ml (= 5 µg/cm²)	–	0.05M HCl	3x PBS	no
Fibronectin	Sigma	–	20 µg/ml (= 5 µg/cm²)	–	PBS	no wash	yes

### Immunohistochemistry

Primary cells were fixed in 4% paraformaldehyde/PBS for 15 minutes and washed thrice with PBS. Next, cells were blocked with blocking buffer (PBS containing 5% donkey serum and 0.25% Triton X-100) for 1h at RT and incubated with primary antibodies diluted in PBS + blocking buffer (1:1) as mentioned in **Table 2** at 4°C overnight. After three PBS washing steps, secondary antibodies (**Table 2**) were applied for 2 h at RT. Nuclei were counterstained with Hoechst, and cover glasses were mounted with Shandon™ Immu-Mount™ (Epredia) and imaged using Nikon Eclipse TE2000-E or Nikon Eclipse Ti2 fluorescent microscopes.

**Table 2:** Antibodies and used dilutions.

Primary Antibodies	Company	Dilution
chicken anti-GFAP	Biolegend	1:1000
goat anti-SOX10	custom-made (aliquot kindly provided by Prof. Michael Wegener, University of Erlangen (26))	1:3000
mouse anti-αSMA	Dianova	1:800 – 1:1000
human anti-ANNA1	kindly provided by the Mayo Clinic (USA)	1:10000
rabbit anti-Connexin 43	custom-made (aliquot kindly provided by Prof. Christian Steinhäuser (27))	1:500

Secondary Antibodies	Company	Dilution
donkey anti-chicken CF633	Sigma Aldrich	1:800
donkey anti-goat Cy3	Dianova	1:800
donkey anti-mouse AF488	Dianova	1:800
donkey anti-human DyLight488	Invitrogen	1:800
donkey anti-rabbit AF647	Southern Biotech	1:800

### Brightfield analysis for area measurement

For Brightfield analysis, cells were fixed in 4% paraformaldehyde/PBS for 15 minutes and washed thrice with PBS. Brightfield imaging was performed using a Nikon Eclipse Ti2 microscope and the Nikon DS-Qi2 camera. For each coating and experiment, three wells were imaged, acquiring large images of 5x5 images at 100x magnification, achieving an overview of the cover glass. Area measurements were done with Fiji software, as described previously (28).

### Enzyme-linked immunosorbent assay

Release of IL-6 and CCL2 was measured in enteric glial cell culture supernatants treated with or without IL-1β for 24h. Supernatants were collected, centrifuged at 500 x g for 5 min, transferred to new tubes, and stored at -20°C before being processed for the IL-6 or CCL2 ELISA. ELISA kits were purchased from Thermo Fisher Scientific and used according to the manufacturer’s instructions.

### Isolation of *in vivo* enteric glial-specific RNA from *muscularis externa*

Enteric glia-specific mRNA from the naive *muscularis externa* of Sox10^iCreERT2^Rpl22^HA/+^ mice was performed as described previously (29). Briefly, small intestines were harvested and *muscularis externa* was mechanically separated from the mucosal layer. *Muscularis* tissue was lysed in two rounds in a Qiagen Powerlyzer24 (2600 rpm, 30 s, 5 min intermediate incubation on ice). Supernatants were incubated with 100 µl HA−coupled A/G beads (Cat.#HY-K0201 MedChemExpress Company) at 4°C and 7 rpm overnight. Magnetic separation was performed for 1 minute, and ribosomes containing mRNA were eluted from beads. RNA was isolated using the Qiagen RNeasy micro kit.

### 3’ bulk mRNA sequencing

RNA sequencing was performed by the Genomics Core Facility of the University Hospital Bonn. For the preparation of libraries, QuantSeq 3’ mRNA-Seq Library Prep Kit (Lexogen) according to the manufacturer’s instructions. Libraries were sequenced on the NovaSeq6000 with a sequencing depth of 10M raw reads. RNA-Seq data were analyzed by PartekFlow^1^ software (USA) using the Lexogen pipeline *12112017*. The pipeline consisted of two adapter trimming and a base-trimming step with subsequent quality controls (QC). Reads were aligned with star2.5.3, followed by a post-alignment QC and quantification to an annotation model. Normalized counts were subjected to a noise reduction filter (excluding features where maximum ≤ 15) followed by principal component and DESeq2 analysis. Sequencing files are deposited in the Gene Expression Omnibus (GEO) database under the GEO accession code GSE271114. Visualization was done with PartekFlow software and GraphPad Prism 10.

### Software

The software tools used for this study include PartekFlow^1^, including DESeq2, PCA, and heatmaps. Venn diagrams were done in PartekFlow or using the bioinformatics web tool^2^. Microscopic images were taken with Nikon NIS-Elements version AR 5.42.04 software and analyzed using ImageJ. Graphs were prepared with GraphPad Prism 10. Graphs and images were combined into figures using Affinity Designer version 2.4.0.

### Statistical analysis

Statistical analyses were performed with Prism 10.0 (GraphPad) using one-way ANOVA or two-way ANOVA with Tukey’s or Šídák’s multiple comparison tests, as stated in the figure legends. In all figures, p-values are indicated as * =p<0.05, ** =p<0.01, *** =p<0.001 and **** =p<0.0001 when compared to poly-L-ornithine as reference or to naive controls as indicated. All plots are mean ± SEM of the indicated numbers of replicates. The PartekFlow software was used to analyze RNAseq data. Herein, statistical analyses were performed using Fisher’s exact test which provided *p*-values with multiple testing corrections (FDR). The significance level for differentially expressed genes was set to FDR<0.05 and indicated in the figure legends were applicable.

## Results

### Matrigel and laminin advance primary enteric glial cell yield and network formation

**Figure 1:**
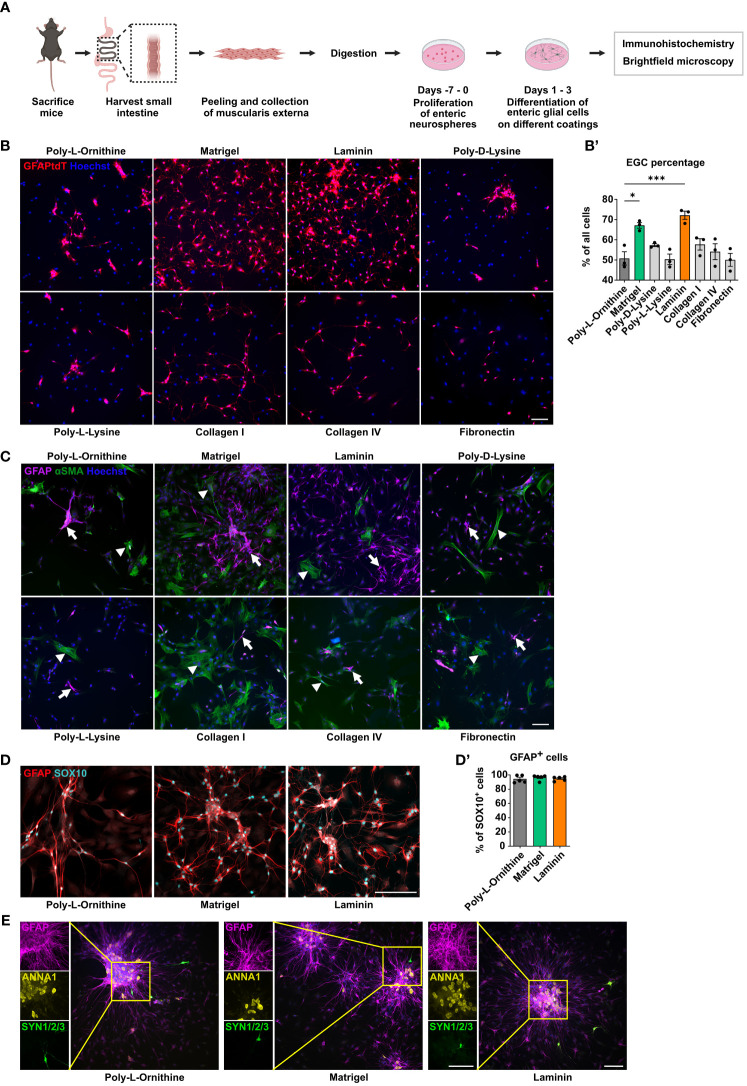
Matrigel and laminin advance primary enteric glial cell yield. **(A)** Schematic representation of the enteric glia culture model. **(B)** Enteric glia were isolated from GFAP^Cre^Ai14^fl/fl^ mice and differentiated on the indicated coating substrates. Representative immunofluorescence images of tdTomato^+^ enteric glia and nuclei (Hoechst) on indicated coatings (scale bar 100 µm). (**B’**) Percentage of tdTomato^+^ enteric glia of all cells (Hoechst) were determined, and data shown as mean ± SEM; significance to poly-L-ornithine (used as a reference) *p < 0.05 and ***p < 0.001 (one-way ANOVA with Tukey’s multiple comparisons test). Data points are from three technical replicates, representative for n = 2 independent experiments. **(C-E)** Enteric glia were isolated from C57BL6 mice and differentiated on the indicated coating substrates (scale bar 100 µm). **(C)** Representative immunofluorescence images of GFAP, αSMA, and nuclei (Hoechst) in enteric glia cultures on the indicated coatings. Arrows show GFAP^+^ glial cells; arrowheads show αSMA^+^ smooth muscle cells. **(D)** Representative immunofluorescence images of GFAP and SOX10 in enteric glia cultures on the indicated coatings. (**D’**) Percentage of GFAP^+^ cells among all SOX10^+^ cells in enteric glial cell cultures. Data show one experiment with 5 technical replicates representative for n = 3 biological replicates. Mean percentage per coating substrate: poly-L-ornithine = 94.52%, Matrigel = 96.79%, laminin = 95.22%. **(E)** Representative immunofluorescence images and corresponding close-up images showing GFAP (magenta), ANNA1 (yellow), and Synapsin 1/2/3 (green) in EGC cultures on indicated coatings.

A rising number of studies utilize primary enteric glial cell cultures to unravel their role in intestinal homeostasis and disease. However, the question of whether coating substrates used for cell adhesion can alter enteric glial biology is poorly understood. In the commonly used method of neurosphere-derived enteric glial cell cultures isolated from the murine small intestine *muscularis externa*, we analyzed their morphological properties on eight coating substrates, namely poly-L-ornithine, Matrigel, laminin, poly-D-lysine, poly-L-lysine, collagen type I, collagen type IV, and fibronectin (**Figure 1A**). First, we studied culture purity by isolating enteric glia from GFAP^Cre^Ai14^fl/fl^ reporter mice, expressing tdTomato in all GFAP^+^ cells. Among the eight tested coating substrates, we found the highest enteric glia purity in laminin coating, with 72% enteric glia, closely followed by Matrigel with 67% (**Figures 1B, B’**). As a reference, we used poly-L-ornithine, a coating substrate widely used in CNS and ENS *in vitro* approaches (7, 30, 31). Accordingly, comparing laminin and Matrigel to poly-L-ornithine (51% purity), we saw significantly higher glia enrichment with these two coatings, while poly-D-lysine, poly-L-lysine, collagen I and IV, as well as fibronectin did not differ much from poly-L-ornithine with culture purities ranging from 50% to 60% (**Figures 1B, B’**).

As enteric glia were isolated from myenteric plexus-derived neurospheres, cultures are likely to contain other stromal cells, e.g. smooth muscle cells or neurons. Indeed, a co-staining of smooth muscle actin (αSMA) and GFAP revealed αSMA expression in some GFAP^-^ cells with a fibroblast-like morphology (**Figure 1C**). With Matrigel and laminin presenting the best glial yield, we validated whether the cultured cells also expressed another prominent glial cell marker, SOX10, keeping poly-L-ornithine as a reference. We found co-expression of GFAP and SOX10 with an overlap of ~95% in all three coating conditions (mean percentage per coating substrate: poly-L-ornithine = 94.52%, Matrigel = 96.79%, laminin = 95.22%; **Figures 1D, D’**). Next, we examined how different coating concentrations, affect EGC cultures. Therefore, we repeated the initially performed experiments, which were done with concentrations either recommended by the manufacturer or commonly used in the literature, and added a 5-fold lower and a 2.5-fold higher concentration for poly-L-ornithine, Matrigel, and laminin (**Table 1**). While no major differences could be observed between the different poly-L-ornithine concentrations, Matrigel showed the lowest EGC percentage and numbers with the highest concentration, while the lowest Matrigel concentration resulted in the highest glia enrichment, even exceeding the recommended concentration although this trend was not significant. For laminin, the standard concentration was significantly better compared to the others regarding EGC percentage and numbers (**Supplementary Figures S1A, A’**). We therefore conclude that ECM protein concentrations can be optimized to improve cell enrichment but the manufacturers’ recommended and commonly used concentrations already are within the optimal range. Therefore, we used the recommended concentrations for our further analyses.

**Figure 2:**
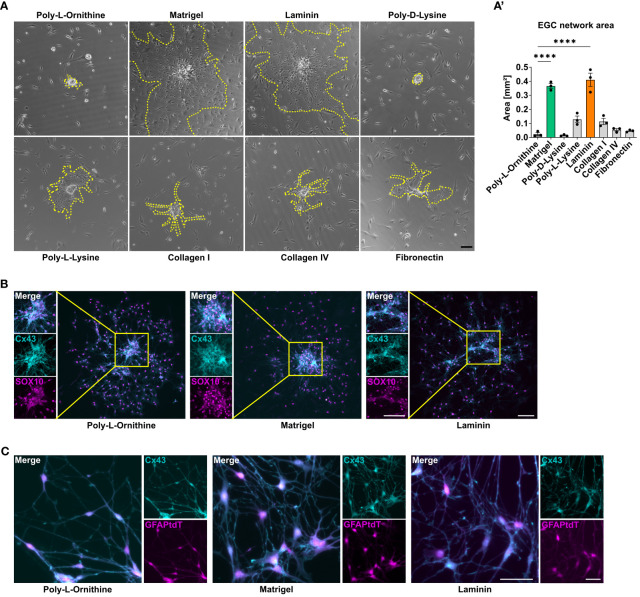
Matrigel and laminin advance primary enteric glial cell network formation. **(A, B)** Enteric glia were isolated from C57BL6 mice and differentiated on different coating substrates. Scale bar 100 µm. **(A)** Representative brightfield images of enteric glia network areas on the indicated coatings. The yellow dotted lines mark the outgrowing glial network arising from one neurosphere. (**A’**) Quantification of enteric glia network area in immunofluorescence images at day three of differentiation on different coating substrates. Data are shown as mean ± SEM and significance to poly-L-ornithine is displayed as ****p < 0.0001 (one-way ANOVA with Tukey’s multiple comparisons test). The data show an experiment with three technical replicates. Five neurosphere network areas were measured for each replicate. Data representative for n = 3 independent experiments. **(B)** Representative immunofluorescence images of connexin (Cx) 43 and SOX10 in enteric glia cultures on the indicated coatings showing Cx43 and SOX10 co-staining and corresponding close-up images of outgrowing sphere regions (scale bar 100 µm). **(C)** Enteric glia were isolated from GFAP^Cre^Ai14^fl/fl^ mice and differentiated on the indicated coating substrates. Representative immunofluorescence images of tdTomato^+^ glia and Cx43 in enteric glia connections (scale bar 50 µm).

Since neurospheres can give rise to enteric glia but also to enteric neurons, we further examined the expression of a specific nuclear neuronal marker, namely ANNA1, as well as synapsins (SYN1/2/3), which label axon terminals. In all three coating conditions, ANNA1^+^ cells were detected, but almost exclusively within the neurospheres but not in the outgrowing cells. SYN1/2/3 was very rarely detected in both, the neurospheres and the outgrown cells, indicating that immature but also differentiated neurons, the latter being able to form synapses were almost absent under all coating conditions (**Figure 1E**).

Microscopical images showed that enteric glia grow out from the neurospheres and form networks, while also individual cells, with no connection to the outgrown network exist. As enteric glia also build functional networks *in vivo*, we were interested if any coating condition might be superior in enabling glial network formation. Therefore, we quantified the enteric glial network area around neurospheres in both brightfield and immunofluorescent images of EGCs on different coating substrates (**Supplementary Figure S1B**). Again, Matrigel and laminin were superior, with a mean glial network area of 0.37 mm^2^ and 0.41 mm^2^ around neurospheres in brightfield images, respectively, which was around 20-fold larger than for poly-L-ornithine coating with 0.02 mm^2^ (**Figures 2A, A’**). Other coating substrates differed slightly from each other but were significantly lower than Matrigel or laminin (poly-D-lysine: 0.01 mm^2^, poly-L-lysine: 0.13 mm^2^, collagen I: 0.1 mm^2^, collagen IV: 0.05 mm^2^, fibronectin 0.05 mm^2^; **Figure 2A, A’**). In order to ensure that it is indeed enteric glia forming these networks, the areas were again measured in tdTomato^+^ cell cultures from GFAP^Cre^Ai14^fl/fl^ reporter mice (**Supplementary Figures S1C, C’**). Similar to the brightfield analysis, Matrigel and lamin surpassed the other coatings also in tdTomato^+^ EGC networks. Comparing the area formation of EGCs on different concentrations of Matrigel, laminin, and poly-L-ornithine, we found similar trends as for EGC percentage and numbers. In detail, no major changes in EGC network area were detected between different poly-L-ornithine concentrations, while Matrigel displayed decreased EGC areas with increasing concentrations. Again, the lowest Matrigel concentration showed a slightly higher potency than the recommended one, but this difference was not significant. For laminin, the standard concentration was surpassing both other concentrations (**Supplementary Figure S1D**).

As connexin-43 (Cx43) hemichannels have been shown to be required for intercellular signal transduction and communication in glial networks *in vivo* (32), we studied the formation of Cx43. Cx43 expression was detected in enteric glia in all coating conditions, both in outgrowing network forming areas and spheres, suggesting that all studied coatings allow functional glial network formation *in vitro* (**Figures 2B, C**).

Together, Matrigel and laminin yielded the highest purity and network formation capacity among the tested ECM substrates and therefore, we performed all following experiments with the standard concentrations of these both coatings in comparison to poly-L-ornithine as a reference.

### Enteric glia on Matrigel and laminin coating display similar transcriptional profiles but differ from poly-L-ornithine

**Figure 3:**
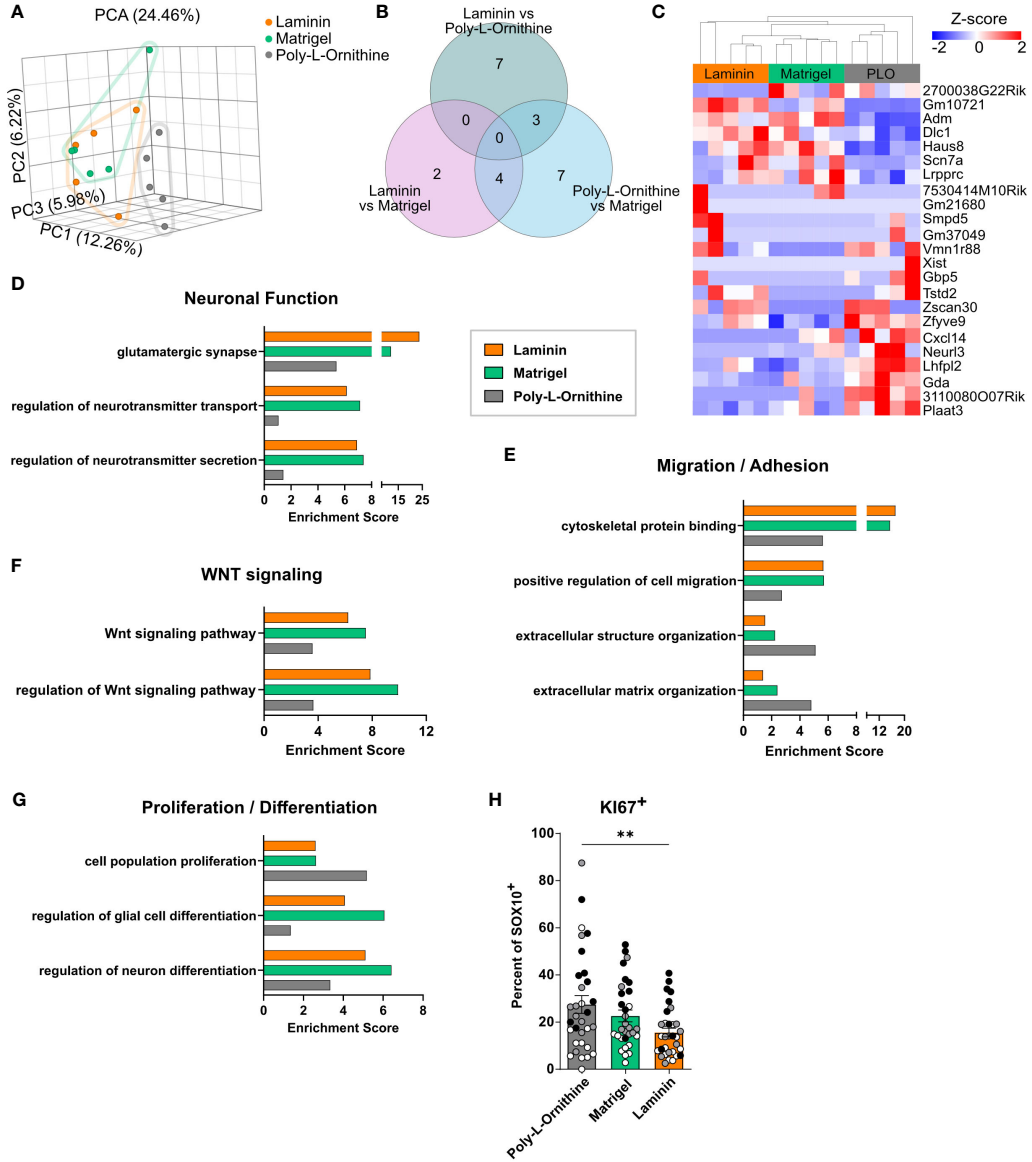
Enteric glia on Matrigel and laminin coating display similar transcriptional profiles but differ from poly-L-ornithine. **(A-G)** Enteric glia were isolated from C57BL6 mice, differentiated on indicated coatings, and processed for bulk 3’ mRNA sequencing (n = 5 biological replicates). **(A)** Principal component analysis (PCA) of enteric glial cell cultures on different coatings. Each dot represents one biological replicate. **(B, C)** Venn diagram **(B)** and heatmap **(C)** showing differentially expressed genes of enteric glia on different coating substrates (FDR < 0.05). PLO = poly-L-ornithine. **(D-G)** Gene set enrichment analysis for genes highly expressed (maximum counts > 15) by enteric glia under naive conditions on poly-L-ornithine (grey), Matrigel (green) and laminin (orange) coating. Selected gene sets are displayed and related to neuronal function **(D)**, migration/adhesion **(E)**, WNT signaling **(F)** and proliferation/differentiation **(G)**. **(H)** Enteric glia were isolated from C57BL6 mice and differentiated on indicated coatings. Percentage of KI67^+^ cells of SOX10^+^ glia were determined in EGC cultures. Data are shown as mean ± SEM and significance is displayed as **p < 0.01 (one-way ANOVA with Tukey’s multiple comparisons test). The data show 10-12 spheres per coating and per biological replicate (n = 3). Dots are colored by biological replicate.

Since the influence of ECM constituents reaches beyond cell morphology, culture purity and cellular network formation, we next analyzed the transcriptomic differences of enteric glia cultured on Matrigel, laminin or poly-L-ornithine. Principal component analysis revealed a separation of enteric glial cultured on poly-L-ornithine from those cultured on Matrigel or laminin, which were very similar (**Figure 3A**). Looking at the number of differentially expressed genes (DEG), we found laminin and Matrigel showing the lowest amount of DEG (6 genes), while poly-L-ornithine differed from Matrigel or laminin by 14 and 10 genes, respectively (**Figure 3B**). Hierarchical clustering of these DEGs confirmed increased expression of genes related to homeostasis (*Adm* (33)), cytoskeletal organization and migration (*Dlc1*, *Haus8*, *Lrpprc*) as well as signal transduction (*Scn7a* (34)) in enteric glia on Matrigel and laminin compared to poly-L-ornithine. Interestingly, recent single-cell RNAseq studies found a strong expression of *Scn7a* in enteric glia and a subset of stromal cells but not in enteric neurons^3^. Genes with higher expression in enteric glia on poly-L-ornithine were related to membrane damage (*Plaat3*) and inflammatory pathways (*Gbp5* (35), *Zfyve9*, *Neurl3*). The latter genes were described as more expressed in enteric glia than enteric neurons^3^. Although typical glial markers like *Sox10*, *Gfap*, *Plp1* or *S100b* were not differentially expressed between the three different coating conditions, DEGs related to homeostasis, signal transduction, and inflammation supported our hypothesis of a difference between Matrigel and laminin versus poly-L-ornithine cultured glia (**Figure 3C**).

To further address the transcriptional pathways activated in enteric glia by different coating substrates, we performed a gene set enrichment analysis of highly expressed genes (maximum counts > 15) for each coating. Here, we found gene sets related to neuronal functions, such as “glutamatergic synapse”, “regulation of neurotransmitter transport”, and “regulation of neurotransmitter secretion” enriched in Matrigel and laminin (**Figure 3D**). As we could only rarely detected neurons in our glial cultures, these enrichments indicate a neurosupportive function of enteric glia and not differentiation of the glia towards a neuronal phenotype. Moreover, gene sets related to “cytoskeletal protein binding” and “positive regulation of cell migration” showed 2-fold enrichment in Matrigel and laminin compared to poly-L-ornithine, suggesting higher regulation of genes related to adhesion and cell motility (**Figure 3E**). On the other hand, enteric glia on poly-L-ornithine showed stronger enrichment of the GO terms “extracellular structure organization” and “extracellular matrix organization”, hinting at an enhanced reaction of enteric glia to their “artificial” environment. Interestingly, gene sets for “Wnt signaling pathway” and “regulation of Wnt signaling pathway” displayed more pronounced enrichment scores in Matrigel- and laminin-treated enteric glia, suggesting functional differences of these cells regarding Wnt-induced neural differentiation processes (36) and Wnt-regulated epithelial homeostasis, a well-known feature of enteric glia (37) (**Figure 3F**). Importantly, gene sets for “regulation of glial cell differentiation” and “regulation of neuron differentiation” were enriched in Matrigel- and laminin-treated glia, while poly-L-ornithine-treated glia exhibited enrichment for the gene set “cell population proliferation” (**Figure 3G**). To further analyze the aspect of proliferation, we quantified the amount of KI67^+^ cells in EGC cultures on different coatings. Indeed, the percentage of KI67^+^ cells among the SOX10^+^ enteric glia was significantly higher in poly-L-ornithine compared to laminin cultures, with a similar trend, although not significant, compared to Matrigel (**Figure 3H**, **Supplementary S2A, B**). Together, this suggests a rather proliferative state of cells on poly-L-ornithine and a more differentiated enteric glia phenotype on Matrigel and laminin coatings.

In conclusion, transcriptomes of enteric glia on different coating substrates revealed differential expression of genes related to homeostasis, migration, adhesion, Wnt signaling and neuronal cell differentiation, identifying Matrigel and laminin as substrates, potentially promoting glial cell differentiation and homeostatic functionality *in vitro*.

### Enteric glial *in vitro* and *in vivo* depict distinct transcriptomic differences

As cell culture models should mimic *in vivo* states, our next goal was to compare (*in vitro*) enteric glia on different coating substrates with their native (*in vivo*) counterparts. To this end, we used Sox10^iCreERT2^Rpl22^HA/+^ (*RiboTag*) mice, which enable glial-cell specific mRNA isolation directly from tissues (29) (**Supplementary Figure S3A**). Principal component analysis of RNAseq data showed clear differences between the *in vivo* and *in vitro* cultured glia (**Supplementary Figure S3B**). PC1-related genes were responsible for the vast difference (28.18%) between all samples. However, disregarding quantitative differences among the top 1000 genes expressed in each condition, we found about 60% overlap between *in vitro* and *in vivo* samples, not differing between the tested coatings (poly-L-ornithine: 60.1%, Matrigel: 60.5%, laminin 60.4%). Overall, 569 of 1000 genes overlapped between all *in vitro* and *in vivo* conditions, hinting at the conservation of ~57% of genes *in vitro*. Interestingly, 369 genes were only expressed in the *RiboTag* group, and 336 genes were exclusively expressed within all *in vitro* conditions (**Supplementary Figure S3C**), highlighting also substantial differences between *in vivo* and *in vitro* enteric glia. To further address these differences, we evaluated the expression of marker genes uniquely expressed by enteric glia (derived from (38)). Although a few *in vitro* samples displayed high expression of single genes (*Aldh1a1*, *Clu*) comparable to the expression in *RiboTag* samples, the vast majority of genes, including *Gfap*, *S100b*, *Sox10,* and *Plp1*, showed a much stronger expression within the *in vivo* enteric glia, with no obvious differences between coating substrates (**Supplementary Figure S3D**). Together, these data suggest the existence of both a conserved genetic profile that is independent of the cellular environment and environmentally dependent genes whose expression differs between cultured and *in vivo* glia. Nevertheless, it should be noted that the transcriptional patterns detected *in vitro* are not uniquely representing enteric glia responses as other contaminating cell types are also included in these cultures. Our comparison with the *Ribotag* approach underlines the need for suitable *in vivo* model systems to study enteric glia biology.

### ECM substrates differentially affect reactive enteric glia transcriptomes after IL-1β activation

**Figure 4:**
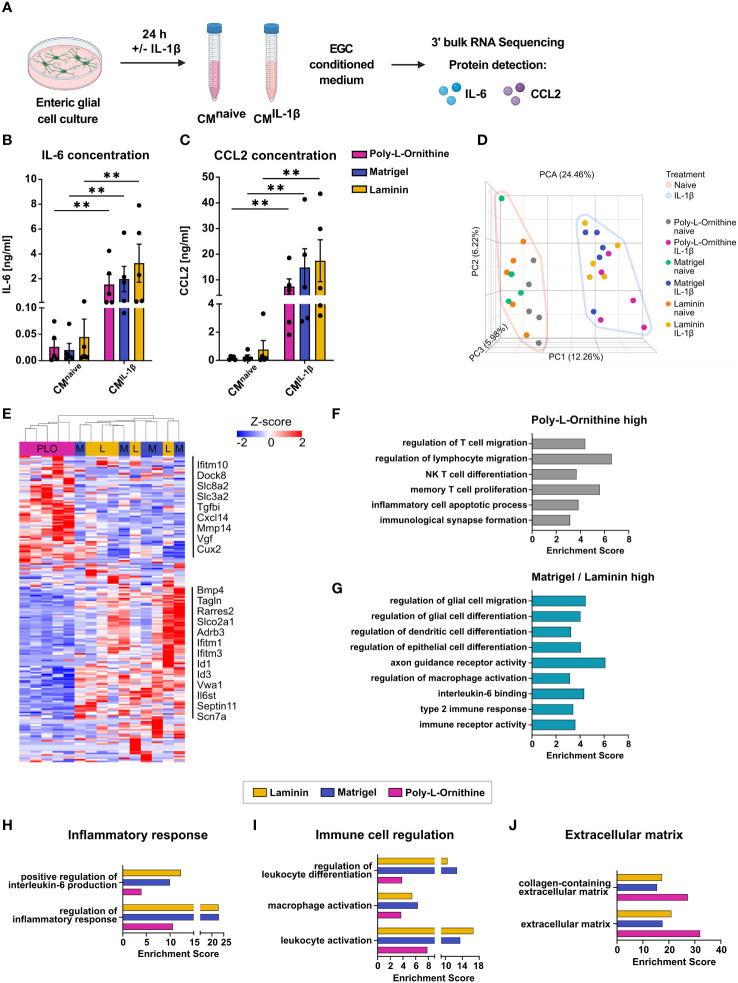
ECM substrates differentially affect reactive enteric glia transcriptomes after IL-1β activation. **(A-J)** Enteric glial cell cultures on poly-L-ornithine, Matrigel or laminin coating were treated with or without IL-1β (10 ng/ml) for 24h. Medium was collected for supernatant protein detection, and cells were processed for bulk 3’ mRNA sequencing. **(A)** Schematic representation of enteric glia IL-1β stimulation. **(B, C)** Protein concentrations of IL-6 **(B)** and CCL2 **(C)** in enteric glia supernatants after stimulation with or without IL-1β on poly-L-ornithine (magenta), Matrigel (blue) or laminin (yellow) coating substrates. Data shown as mean ± SEM; significance to naive displayed as **p < 0.01 (two-way ANOVA with Šídák’s multiple comparisons test). Each dot represents one biological replicate (n = 5). **(D)** Principal component analysis (PCA) of naive and IL-1β-treated primary enteric glia cultures on different coating substrates. Each dot represents one biological replicate (n = 5). **(E)** Heatmap of differentially expressed genes between IL-1β-treated enteric glial cultures. Displayed genes are differentially expressed between coatings (FDR < 0.05). Selected gene names are marked. PLO = Poly-L-ornithine, M = Matrigel, and L = laminin. **(F)** Gene set enrichment analysis of genes upregulated in IL-1β-treated enteric glia on poly-L-ornithine coating compared to IL-1β-treated enteric glia on Matrigel or laminin coatings (FDR < 0.05). **(G)** Gene set enrichment analysis of genes upregulated in IL-1β-treated enteric glia on Matrigel or laminin coatings compared to IL-1β-treated enteric glia on poly-L-ornithine coating (FDR < 0.05). **(H-J)** Gene set enrichment analysis of genes upregulated in IL-1β-treated vs. naive enteric glia for each coating (FDR < 0.05). Displayed gene sets are related to inflammatory response **(H)**, immune cell regulation **(I)**, and extracellular matrix **(J)**.

As enteric glia are of rising interest in inflammatory diseases, we next addressed the effect of coating substrates on enteric glial immune reactivity. Enteric glia were cultured on Matrigel, laminin or poly-L-ornithine and treated with interleukin-1β (IL-1β), a cytokine well-known to induce an immune-reactive state of enteric glia *in vivo* and *in vitro* (**Figure 4A**). *Il1r1* expression in enteric glia was comparable between all coatings under naive conditions and increased by IL-1β treatment in all coatings to the same extent (**Supplementary Figure S4A**). Protein concentration measurements of IL-6 and CCL2 in culture supernatants determined the reactivity of IL-1β-triggered enteric glia. We found significant and comparable increases of IL-6 and CCL2 protein levels in IL-1β-treated enteric glia compared to naive glia for all coatings (**Figures 4B, C**), suggesting that although coating substrates affect enteric glia under naive conditions, their innate immune response regarding IL-6 and CCL2 protein secretion is unaffected by the ECM *in vitro*. This aligns with the overall transcriptional profile, as the principal component analysis of naive and IL-1β-treated enteric glia on different coatings revealed distinct clustering of all naive and all IL-1β-treated samples, with no major differences between different coatings (**Figure 4D**). However, comparing only IL-1β-treated samples, differences between coating substrates became more evident, with poly-L-ornithine again differing from Matrigel and laminin (**Supplementary Figure S4B**).

We found 173 DEGs in IL-1β-triggered enteric glia with no overlap between all three coating substrates. Generally, gene expression was comparable between Matrigel and laminin but differed from poly-L-ornithine (**Figure 4E**, **Supplementary S4C**). Among all DEGs, a subset was upregulated in poly-L-ornithine but not in Matrigel or laminin, which included genes involved in immune cell recruitment and activation (*Ifitm10*, *Dock8*, *Cxcl14*), regulation of homeostatic calcium levels (*Slc8a2*, *Slc3a2*) and cell-collagen interactions (*Tgfbi*, *Mmp17*). Another subset was upregulated in Matrigel and laminin but not in poly-L-ornithine with genes related to glial immune functions and gliogenesis (*Bmp4*, *Tagln*, *Tmem176a*), signal transduction (*Slco2a1*, *Adrb3*, *Il6st*) or interferon-I signaling (*Ifitm1*, *Ifitm3*) (**Figure 4E**). To further resolve potential pathways related to this differential expression pattern, we performed gene set enrichment analysis for genes upregulated in enteric glia on poly-L-ornithine but not on Matrigel and laminin, as well as the other way around (**Figures 4F,G**). Interestingly, pathways enriched in enteric glia on poly-L-ornithine coating were related to T cell immunity and inflammatory processes (**Figure 4F**), while enteric glia on Matrigel or laminin showed enrichment for pathways in context to glial, epithelial and dendritic cell differentiation, as well as regulation of macrophage activation (**Figure 4G**). This suggests a potential functionally diverse immune state of enteric glia dependent on the coating substrate.

To further study the effect of the IL-1β stimulus between the different coatings, we compared IL-1β-treated enteric glia to their respective naive control. Although IL-1β-activation resulted in similar numbers of up- and downregulated genes in all coatings (Matrigel: 349 up, 355 down; laminin: 340 up, 292 down; poly-L-ornithine: 341 up, 353 down), their functional implications differed. To this end, we found stronger enrichment of gene sets related to “regulation of inflammatory response” in enteric glia cultured on Matrigel and laminin than on poly-L-ornithine (**Figures 4H**). Interestingly, although no obvious differences were observed for IL-6 protein levels of enteric glial cultures (**Figure 4B**), the gene set “positive regulation of interleukin-6 production” showed stronger enrichment in laminin and Matrigel, compared to poly-L-ornithine coatings (**Figure 4H**). Confirming a potentially more pronounced involvement of enteric glia on Matrigel and laminin in immune regulation, gene sets related to immune cell activation and differentiation also displayed higher enrichment scores and expression levels (**Figure 4I**, **Supplementary S4D**). Notably, gene sets associated with ECM biology showed higher enrichment scores in enteric glia on poly-L-ornithine compared to Matrigel and laminin, as observed under naive conditions (**Figure 4J**).

Subsumed, IL-1β-triggered immune activation of enteric glial cells resulted in the secretion of proinflammatory mediators and the enrichment of immune regulating pathways in glia on all coating substrates. The transcriptional analysis of enteric glia on Matrigel and laminin compared to poly-L-ornithine coating showed distinct differences regarding their ability in glia-immune cell communication with glia on Matrigel and laminin affecting rather myeloid cells and glial maturation, while poly-L-ornithine affected their ability to act on lymphocyte immunity. Most gene sets related to immune responses were enriched in enteric glia on Matrigel and laminin, suggesting a stronger transcriptional response towards the IL-1β-stimulus with these coating substrates.

Taken together, our study revealed an impact of ECM substrates on both homeostatic and immune-reactive enteric glia *in vitro*. Given the superior action of Matrigel and laminin coatings on glial cell numbers, purity, networking formation, and leukocyte-related immune activity, both conditions are favored over others. Therefore, they should become standard in future studies, allowing researchers to standardize and better compare *in vitro* studies related to enteric glial biology.

## Discussion

Over the last few years, primary enteric glial cultures helped to identify novel interaction pathways of enteric glia with other intestinal cell types, including intestinal epithelial (39) and immune cells, such as macrophages (7). Various *in vivo* studies revealed an immunoactive role of enteric glia in intestinal diseases, and *in vitro* studies added further mechanistic insight into enteric glia-immune cell interactions (7, 9, 11, 13, 40). However, the influence of the non-cellular environment, particularly constituents of the ECM, on enteric glia immune responses needs to be better understood. As the intestinal ECM exhibits a complex architecture and is composed of various constituents, such as different collagens and laminins (41), it has a major impact on cellular functions, including differentiation and migration. While the effects of different ECM constituents on cellular and molecular responses of enteric glia are difficult to study *in vivo* and are mostly limited to immunohistochemical analyses, *in vitro* studies allow proper analyses under homeostasis and inflammatory stimuli.

### Impact of ECM substrates on enteric glia differentiation

Herein, we used primary neurosphere-derived enteric glial cells, which were first allowed to proliferate under floating culture conditions in non-coated plates. Afterwards, cells were allowed to attach to and differentiate on eight different ECM substrates. The coating substrates were chosen based on natural components of the ECM *in vivo* and/or widely used substrates in central or peripheral nervous system cultures and, notably, have been used before in primary enteric glial cultures: poly-L-ornithine (42), Matrigel (43), laminin (44), poly-D-lysine (45), poly-L-lysine (44, 46), collagen I (47), collagen IV (22), and fibronectin (44, 48) although only two ECM components are indeed highly abundant in the intestine, with collagen being most strongly expressed in submucosal and mucosal regions (49) and laminin in the *muscularis externa* (44). Except for Matrigel, all coating substrates are composed of only one type of matrix protein, while Matrigel is a mixture of ECM constituents, namely laminin (60%), collagen IV (30%), and entactin (8%) (43). Our data show that laminin and Matrigel coatings resulted in higher purity and percentage of enteric glia than all other conditions.

The differentiation process of primary enteric glia usually takes several days in murine neurosphere-derived cultures. At differentiation day three, GFAP^+^ glial cells mostly do not express β3-tubulin, a neuronal marker (44) expressed during prolonged culture periods (more than seven days). The same study showed that after 21-24 days, enteric glial cell cultures even display stronger expression of β3-tubulin and induction of synaptic genes, i.e. PSD95, as well as a strong drop in GFAP and SOX10 marker expression. Notably, *in vivo*, enteric glia have the ability to trans-differentiate into neurons (25, 50); possibly, the same mechanisms can also be induced by certain *in vitro* conditions over time. In addition, evidence of glial cell de-differentiation was shown for Schwann cells *in vitro* (51) and increased expression of progenitor markers was found in enteric neural crest-derived cells after co-culture with enteric mesenchymal cells (52). In line, a single-cell study confirmed EGCs as neuronal progenitors *in vitro* (53). Together, these data suggest multi-functional properties of EGCs with the ability to adapt dependent on their *in vitro* environment. In our study, we first determined the percentage and, thereby, purity of glial cells in cultures seeded on different coating substrates, and among all substrates, Matrigel and laminin delivered the highest percentages of glial cell numbers. However, glial cultures are never 100% pure, and it is well established that enteric neurospheres can give rise to enteric neurons and enteric glia during differentiation (17, 44). Interestingly, cocultures of enteric glia and fibroblasts with laminin coating have been shown to support glial differentiation while inhibiting neurogenesis (44). By immunostaining for ANNA1, a pan-neuronal marker not expressed by enteric glia, and synapsin 1/2/3, we confirmed that the outgrowing and network-forming cells are indeed glia, not neurons. A few ANNA1^+^ neurons remain within the core of the differentiating neurosphere, but synapsin-expressing neurons are very rare at this state. A similar observation was previously defined as “neurocore” and was described for CNS neurospheres with detectable neurons only in the sphere’s core at day three of differentiation (54), proving that the aggregation of young neurons initiates this structure. The same mechanism could apply to our ENS cultures. Of note, different enteric glia subsets with differential marker expression have been described *in vivo* (53, 55). Quantifying SOX10 and GFAP signals, we did not observe enteric glial subsets in our *in vitro* cultures. In general, the number of primary cells and culture purity are important aspects of *in vitro* studies. Others have described procedures resulting in a surprisingly high percentage of more than 95% when cells were isolated from mucosal and submucosal regions but not proliferated as neurospheres but as single-cell suspensions (56). However, although these cells express GFAP, they did not form prototypically long extensions but built islets of unconnected cells rather than an interconnected glia network. As the differentiation state of primary cells is equally relevant as their purity, primary enteric glia cultures should also be analyzed for the differentiation features of network formation. The area size of these networks surrounding the neurospheres can be used as an indication of cell differentiation (28). While neurosphere differentiation areas were biggest with Matrigel and laminin coatings, poly-L-ornithine and other coatings displayed only small regions of differentiating cells. In line, transcriptional analysis of enteric glia on Matrigel and laminin revealed enrichment of gene sets related to neuronal cell differentiation and Wnt signaling, both needed for epithelium maintenance and integrity (37). Poly-L-ornithine-treated glia displayed increased numbers of KI67^+^ cells, enrichment of GO terms related to cellular proliferation, and ECM structure and organization. As this suggests a potentially stronger reaction to the ECM environment, one could speculate about a higher need for EGCs on poly-L-ornithine in remodeling or even new production of a suitable ECM environment. Therefore, we propose a direct effect of Matrigel and laminin on enteric glia, promoting differentiation, which resulted in enteric glial network formation on day three of differentiation. On the other hand, poly-L-ornithine, as well as poly-D-lysine, poly-L-lysine, collagen I, collagen IV, and fibronectin displayed comparatively slower differentiation as shown by sphere outgrowth and for poly-L-ornithine, also transcriptionally. Notably, we cannot exclude an effect of the reduced glial cell number and purity in poly-L-ornithine compared to Matrigel and laminin on the transcriptional analysis. However, coating substrates with less differentiation capacity in our study have been widely used at later differentiation stages in other studies, hinting at a slower but not generally compromised differentiation of enteric glia cultured on these substrates. Besides the outgrowth and the morphological observation of network formation, the expression of Cx43 is a prerequisite for enteric glial differentiation and intercellular communication. Cx43 hemichannels are also needed for the promotion of hypersensitivity during inflammation (10, 32) as well as control of neuronal death by glial-released nitric oxide and ATP (57). As we found a clear expression of Cx43, independent of the tested ECM coatings, we conclude that enteric neurosphere-derived glia growing on ECM substrates are, per se, able to form functional networks, with network areas being coating-specific.

Of note, the efficacy of some ECM substrates, such as Matrigel and laminin, was dependent on their concentration, while concentration-dependent differences were weaker in poly-L-ornithine. Generally, the manufacturer´s recommended concentrations yielded preferable results, compared with 2.5-fold higher and 5-fold lower concentrations not significantly improving experimental outcomes. For Matrigel, however, the lower concentration resulted in a non-significant slight enrichment in EGC percentage, numbers and area, suggesting that Matrigel could be used more cost-efficiently with higher dilutions. Still, optimal concentrations in terms of price-performance ratio would need further evaluation. As Matrigel’s primary components are laminin (60%) and collagen IV (30%) (58), it is not unexpected that Matrigel is also a superior coating material, but this has so far never been shown. This finding is of particular interest for coculture experiments as it confirms that enteric glia differentiate well on Matrigel and can, therefore, be used in direct 3D coculture systems with epithelial organoid cultures, which are typically grown in Matrigel. As epithelial organoid cultures have been just stimulated with glial culture supernatants instead of applying direct cocultures (37, 59, 60), this would be a major advantage in studies interested in the direct intercellular communication between primary enteric glia and enterocytes.

### Comparison of *in vitro* and *in vivo* enteric glia

Another important finding of this study is the difference between enteric glia in culture and enteric glia *in vivo*. Using Sox10^iCreERT2^Rpl22^HA/+^ mice, we generated *in vivo* transcriptomes of enteric glia from naive mice and compared these to the ones from primary enteric glial cultures on different ECMs. Among the top 1000 expressed genes, more than 50% overlapped between all samples, while more than 30% were solely expressed *in vivo* or in all *in vitro* conditions. No discernible difference was detected between coating substrates, which is likely due to the strong influence of the glial cell origin (*in vivo* versus *in vitro*) and the accompanying difference. We interpret the strong transcriptional difference due to multiple reasons. One of them is that the *Ribotag* approach delivers glial-specific transcriptomes, while the *in vitro* glial cultures contain further cell types. Another one is the microenvironmental differences between the *in vivo* and *in vitro* settings. Cell types, like neurons, with which glial cells usually interact and gain tissue-specific functionality *in vivo* and the complex extracellular matrix composition are missing in the *in vitro* cultures. However, it remains an open question whether cultured glial cells would become more closely related to *in vivo* glia after being cultured for longer periods and/or in the presence of other cells, typically surrounding them in the gut wall. At least some evidence exists for cocultures of enteric glia, for example, with fibroblasts, that promote glial differentiation (44) and with neurons, where both cell types support each other in cellular maturation (61). Accordingly, we hypothesize that cocultures of enteric glia with enteric neurons, fibroblasts, smooth muscle cells, or resident macrophages might trigger a more *in vivo*-like glial phenotype. Using enteric cell cultures from, e.g. Sox10^iCreERT2^Rpl22^HA/+^ mice would be an elegant tool to study glial-specific reactions within these cocultures.

### ECM substrates impact enteric glial immune reactivity

In the last years, enteric glia have been shown to exert immune-modulatory functions. Triggered by immune mediators like IFN-γ (3), IL-1β (8, 9), noradrenalin (40) or ATP (2), they switch into a “reactive” phenotype during inflammation, releasing immunomodulatory factors like cytokines and chemokines. Some of them have been identified to play a particular role in the pathogenesis of postoperative *muscularis externa* inflammation and colitis (i.e. IL-6 (2, 7, 38, 40), CCL2 (9) and CSF-1 (9, 13)), while others play a particular role in worm infection (i.e. CXCL10 (3)). Besides some of these well-known immune factors, extracellular basement membrane molecules, like laminin or ECM modeling enzymes, have been shown to be an important environmental cue in intestinal immune cell differentiation (23), transmigration (62) and immune tolerance (63). While the ECM composition evidently affects enteric glial biology, we wondered if it also changes their immune response. We stimulated enteric glia cultures on poly-L-ornithine, laminin and Matrigel with IL-1β, a well-established inflammatory mediator inducing enteric glial reactivity *in vitro* and *in vivo* (7, 9). All cultures responded with a strong IL-6 and CCL2 release with a non-significant trend of increased expression of both factors in the laminin-coated group. However, on the transcriptional level, we discovered that poly-L-ornithine-treated cells clustered differently from Matrigel and laminin-treated ones, which clustered together. Interestingly, within the GO terms derived from IL-1β-induced DEGs, glia cultured on laminin and Matrigel showed enrichment in gene clusters regulating glial migration, but also myeloid differentiation, macrophage activation and type 2 immune response. These GO terms are in line with recent literature describing the close proximity of glial cells with intestinal resident and monocyte-derived macrophages (7, 11). Herein, glial cells were not only shown to contribute to macrophage migration and their accumulation around myenteric ganglia (7, 11), but also to shape their differentiation during inflammation (9). Within the poly-L-ornithine IL-1β-stimulated cultures, we found an enrichment of genes related to lymphocyte migration, differentiation and proliferation. While the interaction of lymphocytes and enteric glia is so far less well understood, some studies indicate that T-cells interact with enteric glia, controlling Crohn’s disease-associated myenteric plexitis in humans (64) and T-cell activation by antigen presentation (65). Furthermore, innate lymphoid cell type 3 (ILC3) aggregates were infiltrated by enteric glial projections, which can control ILC3’s IL-22 production in a MyD88-dependent manner (66). Future studies must prove the potential impact of ECM composition on glial cell action regarding different immune cell populations. However, given that the gene expression signatures suggest a more pronounced immune response and effect of glia on leukocytes, particularly macrophages, with laminin and Matrigel coatings, these coatings might be preferable for studies addressing enteric glia-macrophage interactions.

### Overall impact

Subsumed, our findings revealed distinct differences in ECM substrates used for enteric glia cultures. Matrigel and laminin coatings delivered the highest enteric glia purity and the widest cellular network outgrowth. Transcriptional analysis of enteric glia on poly-L-ornithine displayed a decelerated differentiation, while Matrigel and laminin coatings promoted glial support in neuronal differentiation and gene signatures related to cell adhesion and migration. A significant amount of genes was expressed in both *in vivo* and *in vitro* enteric glia, but their expression levels also differed strongly in terms of EGC genes. Notably, the transcriptional *in vivo* and *in vitro* comparison comes with some shortcomings due to the cell culture purity but also the simplicity of the microenvironment *in vitro* compared to the *in vivo* condition. Furthermore, the IL-1β-induced immune reactivity of enteric glia differed between coating substrates and revealed that enteric glia on Matrigel and laminin coatings were transcriptionally more active regarding glial biology and myeloid immune cells, while poly-L-ornithine coating promoted pathways related to T cell immunity. Overall, Matrigel and laminin displayed strong similarities and surpassed poly-L-ornithine, as well as poly-D-lysine, poly-L-lysine, collagen I, collagen IV, and fibronectin in all investigated aspects. Our findings support the use of these superior ECM substrates to allow a better comparison of future studies related to enteric glial biology in homeostasis and neuroinflammation.

## Data availability statement

Sequencing data generated for this study are deposited in the Gene Expression Omnibus (GEO) database under the GEO accession code GSE271114, found here: https://www.ncbi.nlm.nih.gov/geo/query/acc.cgi?acc=GSE271114.

## Ethics statement

The animal study was approved by the state agency for nature, environment and consumer protection (LANUV, AZ 81-02.04.2021.A242). The study was conducted in accordance with the local legislation and institutional requirements.

## Author contributions

LS: Conceptualization, Data curation, Investigation, Methodology, Software, Visualization, Writing – original draft, Writing – review & editing, Formal analysis, Project administration, Validation. RS: Conceptualization, Project administration, Supervision, Writing – review & editing, Methodology. EH: Data curation, Writing – review & editing. SW: Conceptualization, Funding acquisition, Project administration, Resources, Supervision, Writing – original draft, Writing – review & editing, Methodology.

## Footnotes

1
https://www.partek.com/partek-flow/#features.

2
https://bioinformatics.psb.ugent.be/webtools/Venn/.

3
www.gutcellatlas.org.
