# Supplementary material for: Extracellular matrix substrates differentially influence enteric glial cell homeostasis and immune reactivity
Source: Front Immunol. 2024 Jul 25;15:1401751. doi: 10.3389/fimmu.2024.1401751 (PMC11306135; doi:10.3389/fimmu.2024.1401751)
Supplement: Supplementary file 1 [file DataSheet_1.pdf]

Supplementary Material

Supplementary Figures

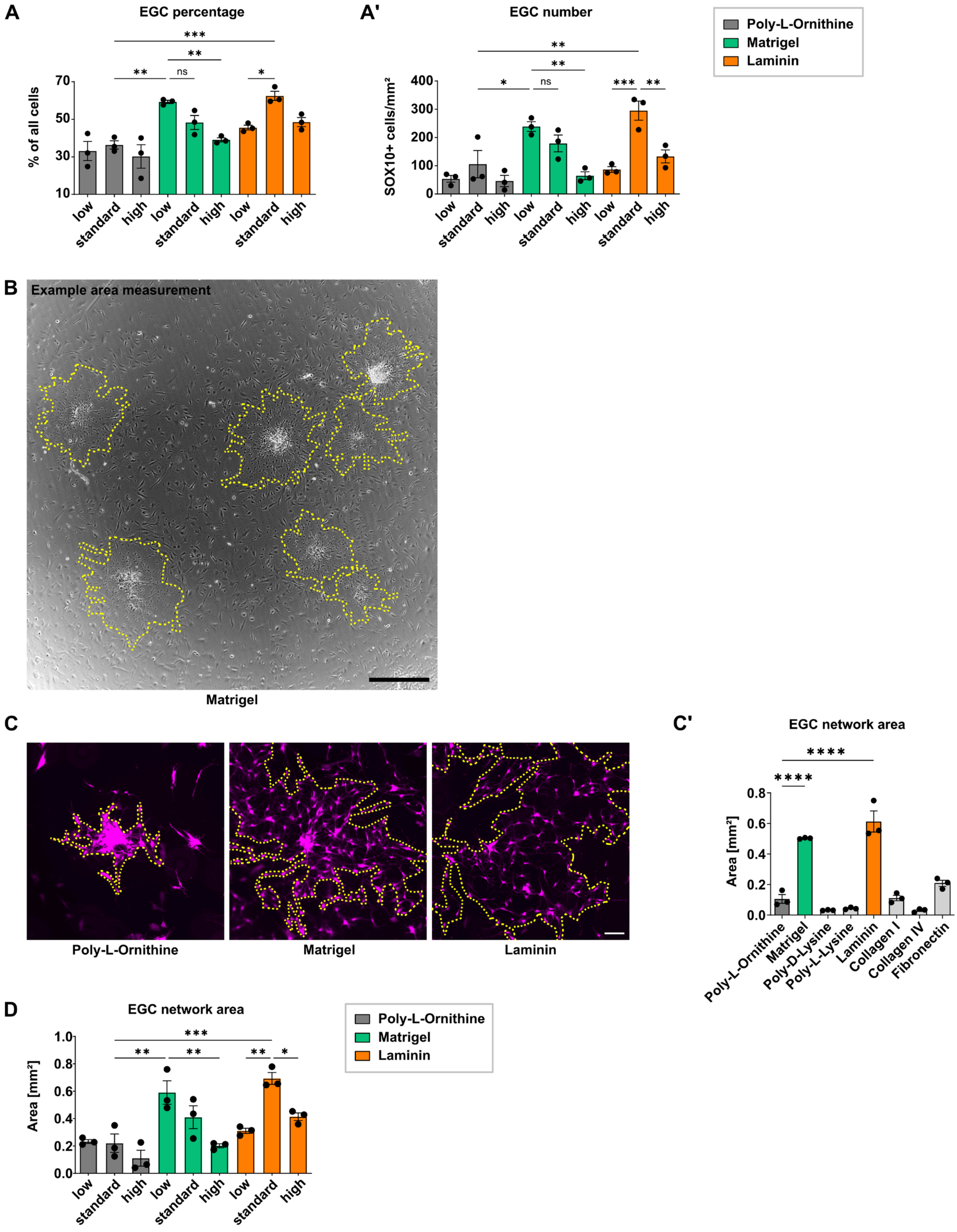

Supplementary Figure 1. Matrigel and laminin advance primary enteric glial cell yield and network formation

(**A-B**) Enteric glia were isolated from C57BL6 mice and differentiated on different coating substrates.
(**A**) Percentage of $SOX10^+$ enteric glia of all cells (Hoechst) and total $SOX10^+$ EGC numbers (**A'**) were determined in EGC cultures on indicated coatings with different concentrations. Data are shown as mean ± SEM and significance is indicated as * p<0.05, **p < 0.01, ***p < 0.001, and ns = not significant (two-way ANOVA with Tukey's multiple comparisons test). Data points are from three biological replicates (n = 3 independent experiments).
(**B**) Representative overview brightfield image of enteric glia on Matrigel coating substrate. The yellow dotted lines display an example area measurement of several outgrowing neurospheres (scale bar 500 µm).
(**C-D**) Enteric glia were isolated from $GFAP^{Cre}Ai14^{fl/fl}$ mice and differentiated on different coating substrates.
(**C**) Representative immunofluorescence images of the outgrowing network areas (yellow dotted lines) of $tdtomato^+$ enteric glia (scale bar 100 µm). (**C'**) Quantification of enteric glia network area at day three of differentiation on the indicated coating substrates. Data show mean ± SEM; significance to poly-L-ornithine is displayed as ****p < 0.0001 (one-way ANOVA with Tukey's multiple comparisons test). The data show an experiment with three technical replicates. Five neurosphere network areas were measured for each replicate. Data are representative for n = 3 independent experiments.
(**D**) Quantification of enteric glia network area at day three of differentiation on different coating substrates with different concentrations. Data are shown as mean ± SEM and significance is indicated as * p<0.05, **p < 0.01, and ***p < 0.001 (two-way ANOVA with Tukey's multiple comparisons test). Five neurosphere network areas were measured per technical replicate, and three technical replicates were analyzed per biological replicate. Data points present biological replicates (n = 3 independent experiments).

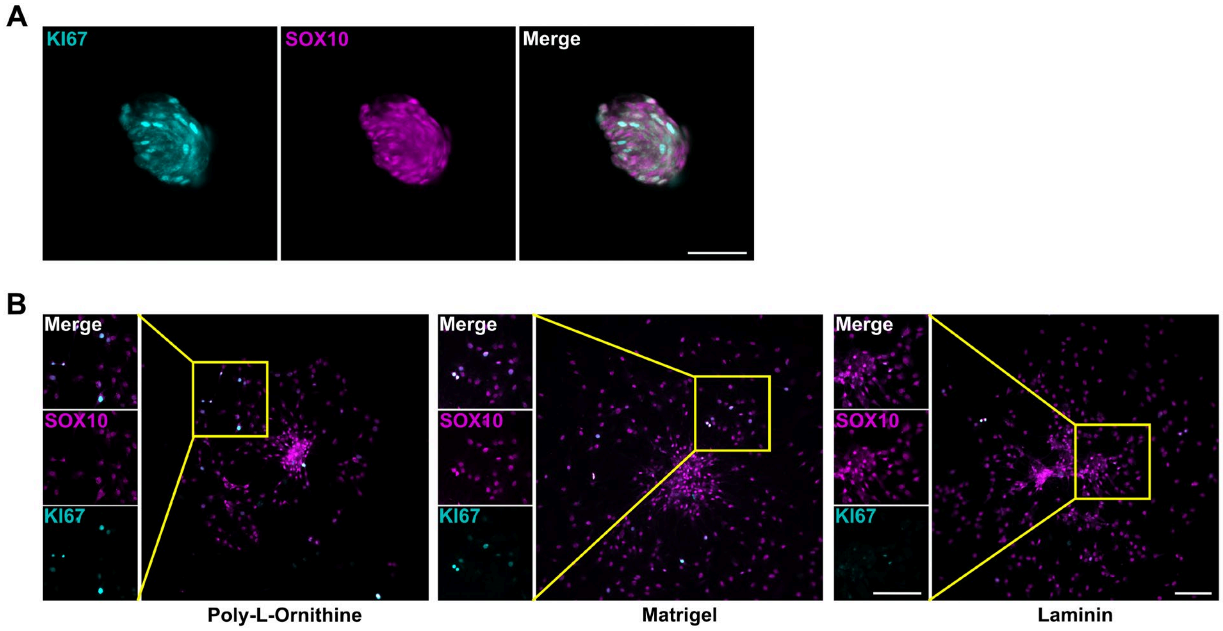

Supplementary Figure 2. Proliferation of enteric glial cell cultures might be coating-dependent

Enteric glia were isolated from C57BL6 mice.
(**A**) Representative immunofluorescence images of KI67 and SOX10 in enteric neurosphere cultures in proliferation medium on day 6 of proliferation (scale bar 50 µm).
(**B**) Representative immunofluorescence images and corresponding close-up images of KI67 and SOX10 in enteric glia cultures on indicated coatings (scale bar 100 µm).

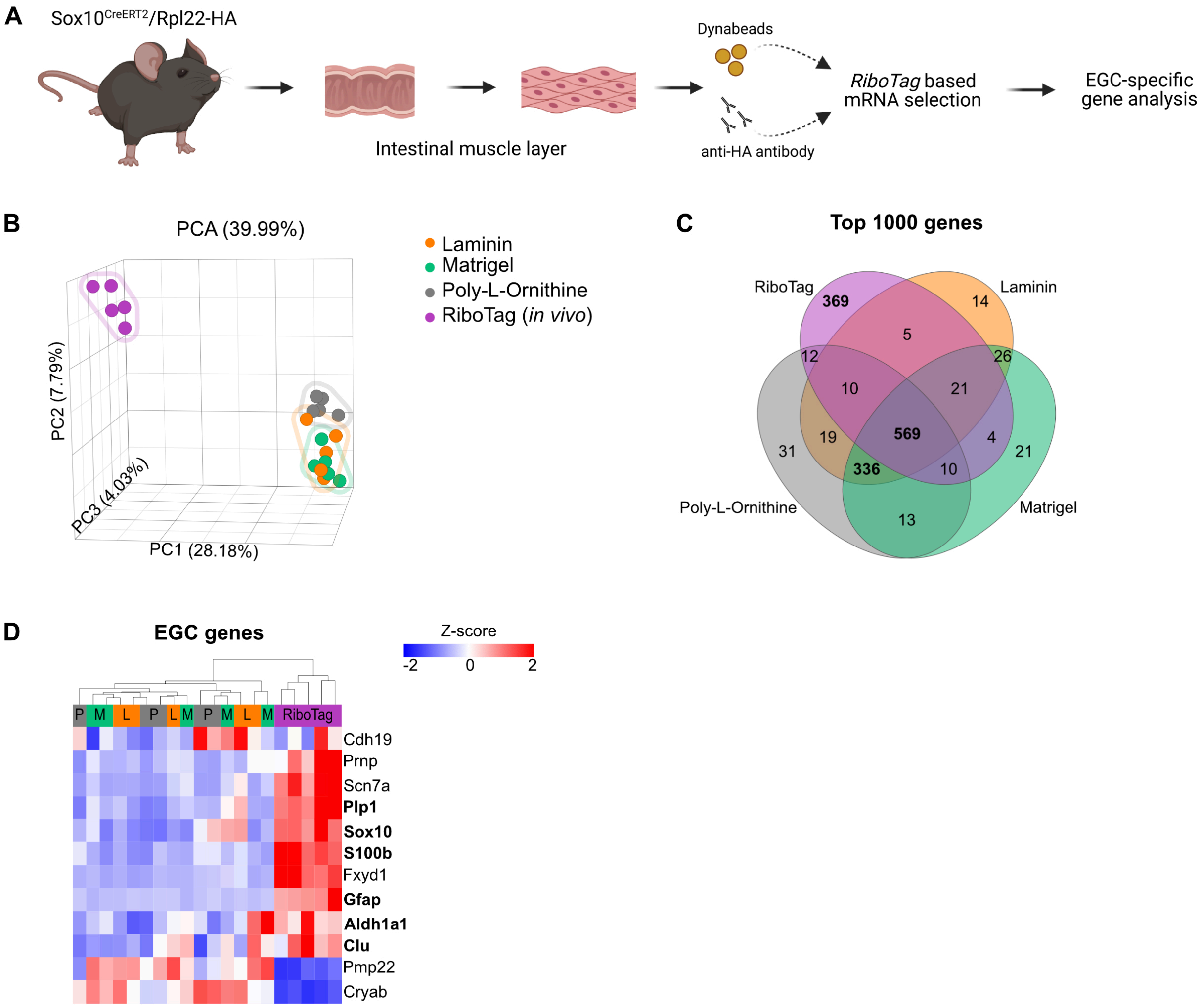

Supplementary Figure 3: Enteric glia *in vitro* and *in vivo* depict distinct transcriptomic profiles

Enteric glial mRNA was isolated from Sox10^{iCreERT2}Rpl22$^{HA/+}$ mice using the *RiboTag* approach. Cell-specific mRNA was processed for bulk 3' mRNA sequencing, and transcriptional profiles were compared with primary enteric glia cultures on indicated coatings.
(**A**) Schematic representation of the *RiboTag* approach.
(**B**) Principal component analysis (PCA) of *RiboTag* samples and primary enteric glia cultures on different coating substrates. Each dot represents one biological replicate (n = 5).
(**C**) Venn diagram displaying overlaps of the top 1000 genes expressed by *in vivo* (*RiboTag*) and *in vitro* enteric glia on poly-L-ornithine (P), Matrigel (M), or laminin (L) coating.
(**D**) Heatmap of enteric glia genes of *in vivo* (*RiboTag*) and *in vitro* enteric glia on poly-L-ornithine (P), Matrigel (M), or laminin (L) coating.

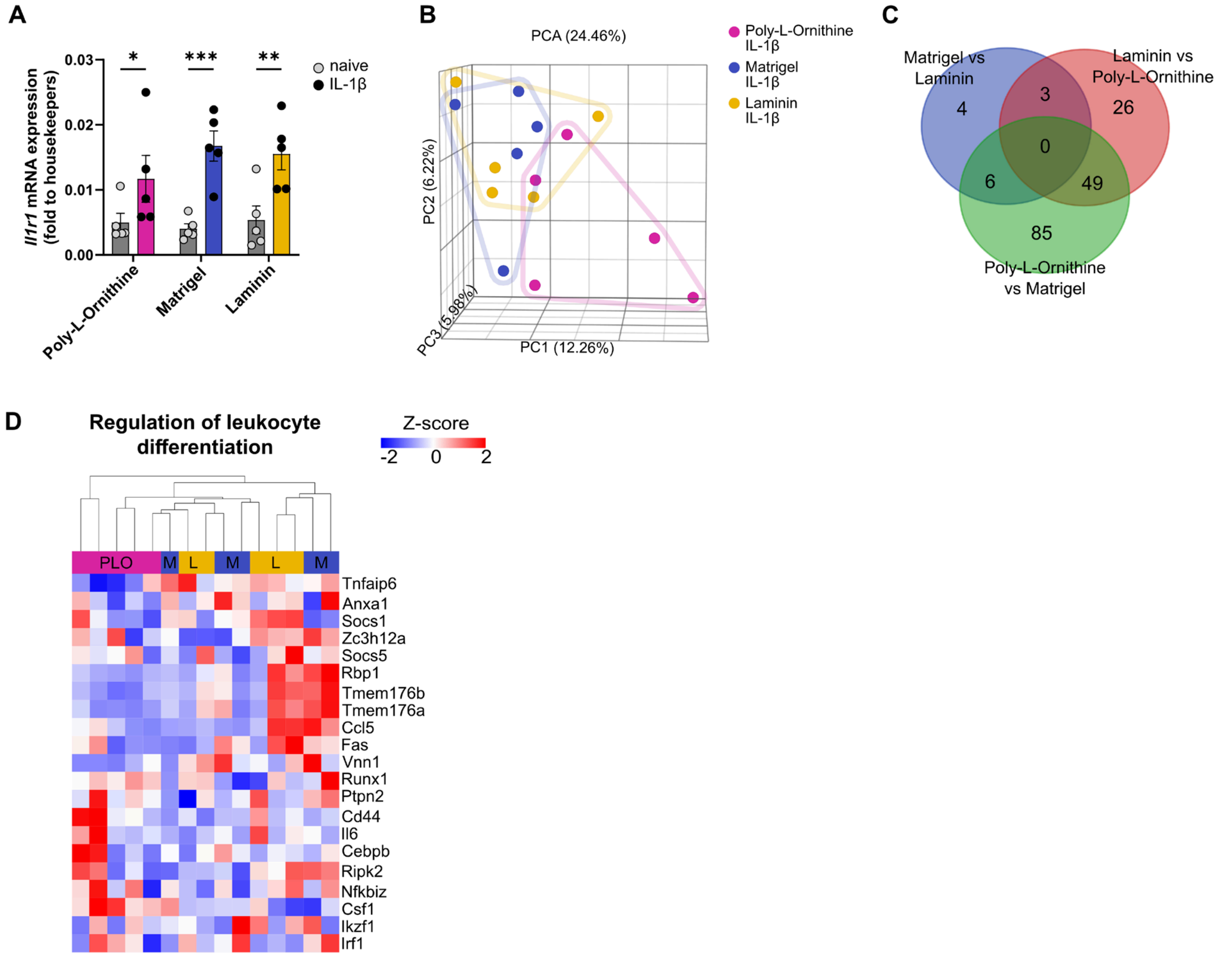

Supplementary Figure 4. ECM substrates differentially affect reactive enteric glia transcriptomes after IL-1β activation

(**A-D**) Enteric glial cell cultures on poly-L-ornithine, Matrigel, or laminin coating were treated with or without IL-1β (10 ng/ml) for 24h and processed for bulk 3' mRNA sequencing.
(**A**) *Il1r1* expression of naive and IL-1β-treated enteric glia on different coatings. Expression levels were derived from sequencing counts and normalized to the mean of 4 housekeeping genes (*Gapdh*, *Rpl32*, *Actb*, *Pgk1*). Data show mean ± SEM of n = 5; significance displayed as *p<0.05, **p<0.01 and ***p<0.001 (two-way ANOVA with Tukey's multiple comparisons test).
(**B**) Principal component analysis (PCA) of IL-1β-treated primary enteric glial cultures on indicated coating substrates. Each dot represents one biological replicate (n = 5).
(**C**) Venn diagram of differentially expressed genes between IL-1β-treated enteric glial cultures. Displayed genes are differentially expressed between coatings (FDR < 0.05).
(**D**) Heatmap of genes derived from the GO term "regulation of leukocyte differentiation". Displayed genes were upregulated after IL-1β treatment in enteric glia on poly-L-ornithine (PLO), Matrigel (M), and/or laminin (L) coating compared to their respective naive controls.
